# 

**Published:** 1884-12

**Authors:** 


### CONTENTS.

COMMUNICATIONS.

Improvement in Artificial Dentures.......... 581
The Nutritive Functions.......... 585
Food—Animal and Vegetable.......... 598

PROCEEDINGS.

Ohio State Dental Society.......... 606

SELECTIONS.

Mucoid or Puruloid Engorgement of the Antrum.......... 608
Oil of Cloves to Remove the Antipathy Against Chloroform.......... 611
SALMAGUNDI.......... 612

EDITORIAL.

Meeting of the Ohio State Dental Society.......... 618
A Syllabus of Lectures on Odontology.......... 619
Commencement.......... 621
Bibliographical.......... 622
Dental Caries.......... 623
A Complete Index to the Dental Cosmos.......... 624
A New Crown.......... 624
End of the Volume.......... 625
Wanted.......... 625
Obituary.......... 626

